# Are Australian podiatrists meeting best practice in the management of diabetic foot complications?

**DOI:** 10.1186/1757-1146-8-S2-O33

**Published:** 2015-09-22

**Authors:** Tom R Quinton, Peter A Lazzarini, Frances M Boyle, Anthony W Russell, David G Armstrong

**Affiliations:** 1 Department of Prosthetics, Orthotics, & Podiatry, Princess Alexandra Hospital, Brisbane, Australia; 2 School of Population Health, The University of Queensland, Brisbane, Australia; 3 Allied Health Research Collaborative, Metro North Hospital & Health Service, Queensland Health, Brisbane, Australia; 4 School of Clinical Sciences, Queensland University of Technology, Brisbane, Australia; 5 Department of Diabetes & Endocrinology, Princess Alexandra Hospital, Brisbane, Australia; 6 School of Medicine, The University of Queensland, Brisbane, Australia; 7 Department of Surgery, Southern Arizona Limb Salvage Alliance (SALSA), University of Arizona College of Medicine, Tucson, United States

**Keywords:** Diabetes, Foot, Ulcer, Australia

## Background

Diabetic foot complications are the leading cause of lower extremity amputation and diabetes-related hospitalisation in Australia. Studies demonstrate significant reductions in amputations and hospitalisation when health professionals implement best practice management. Whilst other nations have surveyed health professionals on specific diabetic foot management, to the best of the authors' knowledge this appears not to have occurred in Australia. The primary aim of this study was to examine Australian podiatrists' diabetic foot management compared with best practice recommendations by the Australian National Health Medical Research Council.

## Methods

A 36-item Australian Diabetic Foot Management survey, employing seven-point Likert scales (0=Never; 7=Always) to measure multiple aspects of best practice diabetic foot management was developed. The survey was briefly tested for face and content validity. The survey was electronically distributed to Australian podiatrists via professional associations. Demographics including sex, years of professional experience, employment-sector and patient numbers were also collected. Chi-squared and Mann Whitney U tests were used to test any differences between sub-groups.

## Results

Three hundred and eleven podiatrists responded; 222 (71%) were female, 158 (51%) from the public sector and 11-15 years median experience. Participants reported treating a median of 21-30 diabetes patients each week, including 1-5 with foot ulcers. Overall, participants registered median scores of at least “very often” (>6) in their use of most items covering best practice diabetic foot management. Notable exceptions were: “never” (1(IQR) (1 – 3)) using total contact casting, “sometimes” (4 (2 – 5)) performing an ankle brachial index, “sometimes” (4 (1 – 6)) using University of Texas Wound Classification System, and “sometimes” (4 (3 – 6) referring to specialist multi-disciplinary foot teams. Public sector podiatrists reported higher use or access on all those items compared to private sector podiatrists (p < 0.01).

## Conclusion and clinical relevance

This study provides the first baseline information on Australian podiatrists' adherence to best practice diabetic foot guidelines. It appears podiatrists manage large caseloads of people with diabetes and are generally implementing best practice guidelines recommendations with some notable exceptions. Further studies are required to identify barriers to implementing these recommendations to ensure all Australians with diabetes have access to best practice care.

